# Synthesis of Low-Cost and High-Performance Dual-Atom Doped Carbon-Based Materials with a Simple Green Route as Anodes for Sodium-Ion Batteries

**DOI:** 10.3390/molecules28217314

**Published:** 2023-10-28

**Authors:** Bin Lu, Chi Zhang, Ding-Rong Deng, Jian-Chun Weng, Jia-Xi Song, Xiao-Hong Fan, Gui-Fang Li, Yi Li, Qi-Hui Wu

**Affiliations:** 1 Xiamen Key Laboratory of Marine Corrosion and Smart Protective Materials, School of Marin Equipment and Mechanical Engineering, Jimei University, Xiamen 361000, China; lb5868933@163.com (B.L.); gingerwjc@163.com (J.-C.W.); sjx-0415@jmu.edu.cn (J.-X.S.); echo_fan@jmu.edu.cn (X.-H.F.); guifangli1991@163.com (G.-F.L.); qihui.wu@jmu.edu.cn (Q.-H.W.); 2 School of Energy and Chemical Engineering, Xiamen University Malaysia, Jalan Sunsuria, Bandar Sunsuria, Sepang 43900, Malaysia; cme2009109@xmu.edu.my; 3 Jiangsu Key Laboratory of Advanced Functional Polymer Design and Application, College of Chemistry, Chemical Engineering and Materials Science, Soochow University, Suzhou 215123, China

**Keywords:** low-cost, sulfur doping, carbons, sodium-ion batteries, anode materials

## Abstract

Sodium-ion batteries (SIBs) are promising alternatives to replace lithium-ion batteries as future energy storage batteries because of their abundant sodium resources, low cost, and high charging efficiency. In order to match the high energy capacity and density, designing an atomically doped carbonous material as the anode is presently one of the important strategies to commercialize SIBs. In this work, we report the preparation of high-performance dual-atom-doped carbon (C) materials using low-cost corn starch and thiourea (CH_4_N_2_S) as the precursors. The electronegativity and radii of the doped atoms and C are different, which can vary the embedding properties of sodium ions (Na^+^) into/on C. As sulfur (S) can effectively expand the layer spacing, it provides more channels for embedding and de-embedding Na^+^. The synergistic effect of N and S co-doping can remarkably boost the performance of SIBs. The capacity is preserved at 400 mAh g ^−1^ after 200 cycles at 500 mA g^−1^; more notably, the initial Coulombic efficiency is 81%. Even at a high rate of high current of 10 A g^−1^, the cell capacity can still reach 170 mAh g^−1^. More importantly, after 3000 cycles at 1 A g^−1^, the capacity decay is less than 0.003% per cycle, which demonstrates its excellent electrochemical performance. These results indicate that high-performance carbon materials can be prepared using low-cost corn starch and thiourea.

## 1. Introduction

With the continuous over-consumption of fossil energies and the emission of greenhouse gases posing a huge threat to the global environment, there is an increasing demand for creating new environmentally friendly high-quality storage systems and low-cost applications to new energy systems [1]. Since 1970, lithium-ion batteries have been leading the way in secondary batteries and are widely used as a power source for electric vehicles because of the high working voltage and superior cycling stability [2]. However, the low reserves of lithium on the Earth and its unequal global distribution have led to an increase in price [3]. Because of the similar physiochemical properties of Na and lithium, sodium is more widely distributed than lithium and has a low production cost, and sodium-ion batteries (SIBs) are potentially promising for mass storage [4,5]. Graphite can achieve excellent electrochemical performance as a lithium-ion battery anode. As the radius of sodium ions (Na^+^) (0.113 nm) is 35% wider than that of lithium ions (0.076 nm), when graphite is used as anodes for SIBs, the diffusion of Na^+^ is impeded during charging and discharging processes, which also leads to expansion of the electrode volume [6]. At present, SIBs anodes are mainly classified into alloys [7], metal oxides [8,9,10], metal sulphides [11], carbons [12], and phosphates [13]; in addition, the metal–organic frameworks also need extensive research [14,15]. However, when considering cycling stability and cost-effectiveness, carbon-based materials have undoubtedly become the most suitable materials. More importantly, biomass-derived carbon materials have a variety of natural advantages when it comes to the sustainability of energy storage and conversion and have proven to be one of the ideal anode materials [16]. Starch, which is the most common biomass in daily life and the most popular polysaccharide, is often used as an emulsifier in food products. Since the microstructure of starch after pyrolysis is an amorphous and layered structure, the abundance of defects plays a positive role in the shuttling of Na^+^. Moreover, because of its low cost, it can be commercialized as anodes for storage batteries [17]. However, the hard carbon produced by the direct pyrolysis of untreated biomasses does not substantially improve the performance of SIBs [18,19]. In order to improve the performance of batteries, heteroatom doping has become one of the most common and effective means in recent years. Because heteroatom doping can effectively change the electron distribution around and on the surface of the carbon atoms, it can generate more electrochemically active sites. It can play a positively significant role in the transport diffusion of Na^+^ and the transfer of electrons, thereby improving the capacity of the SIBs [20]. In recent years, there have been many examples of heteroatom doping carbon, such as the addition of nitrogen, which can create more defects, add more active sites, and effectively increase conductivity [21]. The addition of phosphorus can lead to an increase in electrochemical adsorption/desorption sites, which can accelerate the reaction rate and enhance the capacity of sodium-ion batteries [22]. The addition of elemental sulphury can effectively expand the layer spacing, enabling more sodium ions to pass through per unit of time, and contributing to the rate capacity of the battery [23]. There are also halogen elements doped to improve the performance of sodium-ion batteries [24]. However, most of them are doped with single atoms at present. For dual-atom co-doping, most studies focus on N, O [25], and N, P [26], and there are very few previous reports on (N, S)-co-doped carbon materials that have been applied as anode materials in SIBs [27].

In this paper, we used low-cost corn starch as a carbon source and CH_4_N_2_S as the N and S elemental sources. Carbon materials with high-performance disorder were synthesized using hydrothermal oxidation and high-temperature pyrolysis, which were then used as anodes for SIBs. Following the oxidation of nitric acid, the carbon material produces oxygen-containing functional groups (C-OOH), which makes it easier to dope N and S elements into the carbon-based material. S element can effectively expand the interlayer spacing. Because the electronegativity of N is larger than that of C, its radius is smaller than that of C. After N doping, more defects and active sites are formed, which causes the carbon material to become more disordered and provides more channels for Na^+^ transport. (N, S)-C (N and S doped carbon) has a large specific surface area and stable electrochemical properties, and its highest capacity can reach 620 mAh g^−1^ at 50 mA g^−1^. Even at the high current rate of 10 A g^−1^, the capacity can reach 170 mAh g^−1^. Because of the simplicity and low cost of the production process, it can be commercially produced on a large scale.

## 2. Results and Discussion

**Figure 1:**
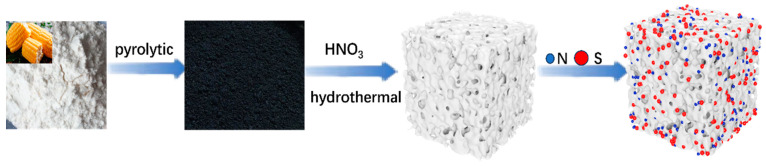
Schematic representation of starch biowaste-derived origin carbon (OC) and N, S-doped carbon ((N, S)-C) obtained via a facile carbonization process.

Figure 1 illustrates the process of (N, S)-C preparation. First, the starch was carbonized at 1100, 1150, and 1190 °C, and then hydrothermal oxidation of the OCs was carried out with concentrated nitric acid; finally, N and S atom doping was performed with carbonization of the OCs in the presence of CH_4_N_2_S.

**Figure 2:**
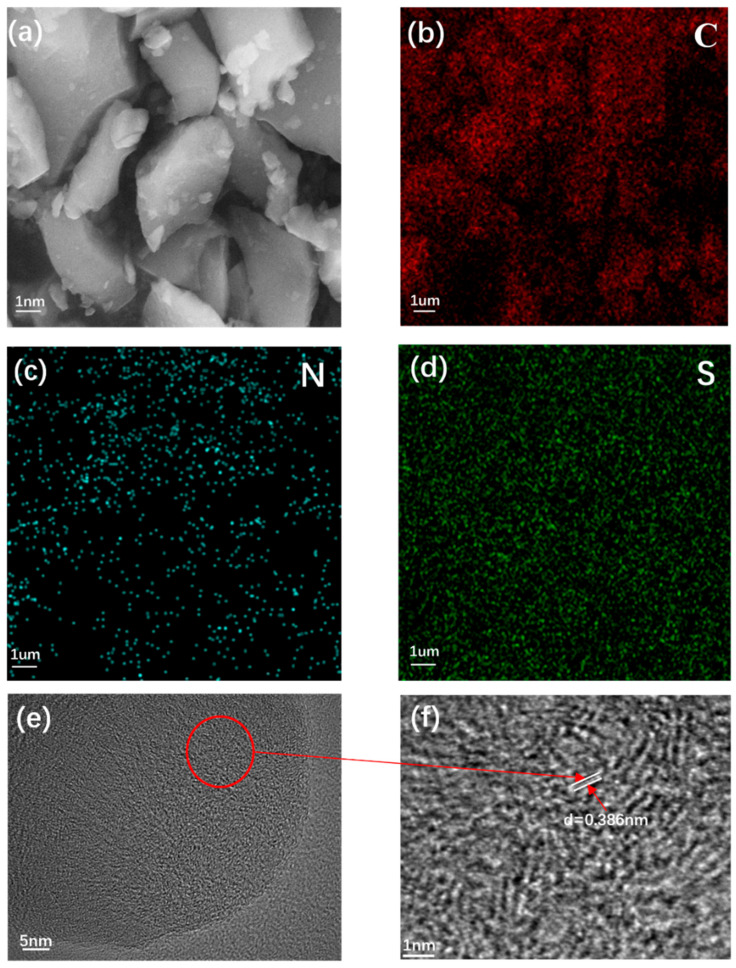
(**a**) SEM images; (**b**–**d**) the EDS elemental mapping of (N,S)-C; (**e**,**f**) TEM images of (N, S)-C.

The structural morphology of the (N, S)-C samples was characterized and is shown in Figure 2. Figure 2a shows that the material exhibits agglomerated particles with diverse sizes, which allows the electrolyte to completely enter the surface of the active material, shortens the distance of Na^+^ transport, and therefore reduces the loss of capacity during diffusion at high current densities. The element mapping images reveal that C, N, and S elements are evenly distributed on the (N, S)-C surface (Figure 2b–d). However, it was difficult to distinguish between (N, S)-C and OC because of their similar morphologies (Figure S1). Therefore, TEM tests were conducted, Figure 2f is a partial enlargement of the circled red area in Figure 2e as displayed; the lattice view of TEM shows the microstructure of the material, and the average interlayer spacing of the lattice stripes was 0.386 nm in (N, S)-C and 0.360 nm in OC (Figure S1), indicating that the layer spacing is clearly enlarged after N and S doping. In addition, the structures of OC and (N, S)-C are predominantly amorphous and abundant in defects of disorder, which are more favorable for Na^+^ storage.

**Figure 3:**
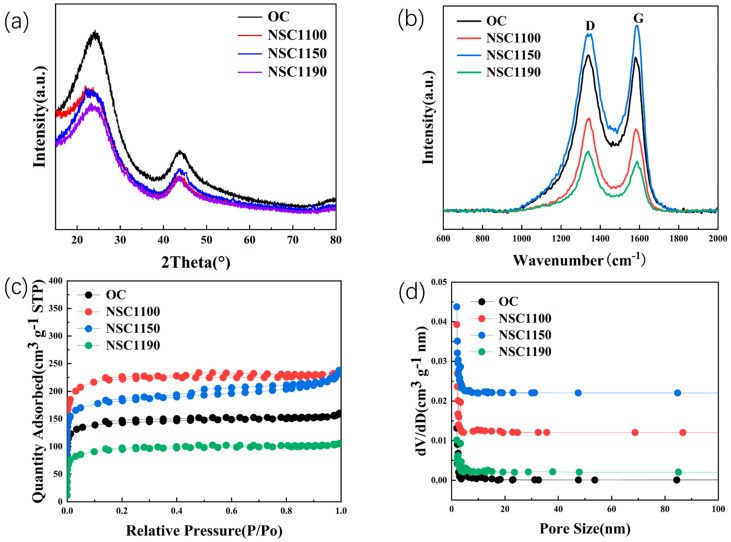
(**a**) XRD patterns and (**b**) Raman spectra of OC and (N, S)-C. (**c**) Nitrogen isothermal adsorption isotherm curves; (**d**) the pore size distributions of OC and (N, S)-C. The peaks of D represents disorder. The peaks of G represents graphitic carbon.

The XRD patterns of (N, S)-C and OC are shown in Figure 3a. Both patterns show peaks at 2θ, approximately at 23° and 43°, corresponding to C peaks (002) and (100). The (002) peak has a higher intensity, indicating a higher crystallinity. More worthy of attention is the (002) peak after N and S co-doping shift to a lower degree, indicating that the interlayer spacing is significantly enlarged after doping, which is consistent with the TEM results [28]. According to the Bragg Equation 2dsinθ = nλ, the (N, S)-C1150 doped layer spacing (d_002_) is 0.386 nm, which is much larger than that of OC. This is helpful for Na^+^ insertion of de-embedding into/from the layer space [29]. However, as the carbonization temperature increases, (N, S)-C1190 is offered to a higher diffraction angle, increasing the graphitization and the layer spacing decreasing to 0.380 nm, but the spacing is still much larger than that of graphite (0.335 nm), allowing Na^+^ to pass through smoothly. The Raman spectra of OC and (N, S)-C, as shown in Figure 3b, exhibit sharp peaks at 1340 and 1580 cm^−1^, which correspond to peaks D and G. The D peak represents disordered or defective grey carbon, indicating the existence of more irregularly shaped carbon. The G peak represents graphitic carbon, indicating the presence of more sp^2^ hybridized carbon. In addition, the degree of carbon disorder was evaluated by measuring the ratio (I_D_/I_G_) of the D and G bands after doping [30]. Among them, the I_D_/I_G_ of (N, S)-C1100, (N, S)-C1150, and (N, S)-C1190 are 1.03, 1.21, and 0.94. The larger ratios indicate that the carbon is more disordered, and that the defects, edges, and structural distortions are increasing. In particular, the ratio of (N, S)-C1150 is as high as 1.21, indicating that it exhibits high disorder due to the doping of N and S. They also favor the presence of N and S atoms on the defects or edge sites of graphite crystals. All the ratios after doping are larger than the undoped ones, which means that due to the doping of N and S elements, it creates more edge defects, provides more active potential sites for Na^+^ adsorption/desorption, and therefore promotes capacity [31].

The specific surface area and pore size were tested using N_2_ adsorption isothermal measurements. As shown in Table 1, the specific surface areas of (N, S)-C1100, (N, S)-C1150, and (N, S)-C1190 are 563.45, 683.85, and 296.67 m^2^ g^−1^, respectively. On the one hand, the decrease in the surface area is mainly due to the closure of some micropores, resulting in a lower specific surface area. Moreover, the surface area increases and then decreases; it was also demonstrated that the greater the number of micropores, the greater the degree of disorder in the carbon material, thereby resulting in a higher I_D_/I_G_ value. All the data are matched using Raman spectroscopy. On the other hand, the increase in the specific surface area may be attributed to the fact that more functional groups (C-OOH) and N and S heteroatoms are doped into the carbon pores [32]. According to IUPAC (International Union of Pure and Applied Chemistry), hysteresis loops can be classified into four types. Figure 3c shows that the OC hysteresis loop conforms to type I, indicating the presence of more micropores. (N, S)-C1100 and (N, S)-C1150 belong to the mixed form of types I and IV, suggesting that the material may be hierarchical and that more mesopores are present [33]. Moreover, the Barrett–Joyner–Halenda (BJH) pore size distributions further confirm abundant microporous and mesoporous pores. Figure 3d shows that most of the pore sizes of the carbon materials are mainly distributed in the range of 0.01–30 nm. The pore size of (N, S)-C1150 is 3.99 nm, and the concentration of the pore sizes promotes rapid transfer at the positive pole and negative pole interface. It is more suitable for Na^+^ shuttling and improves the capacity of the battery [34].

**Figure 4:**
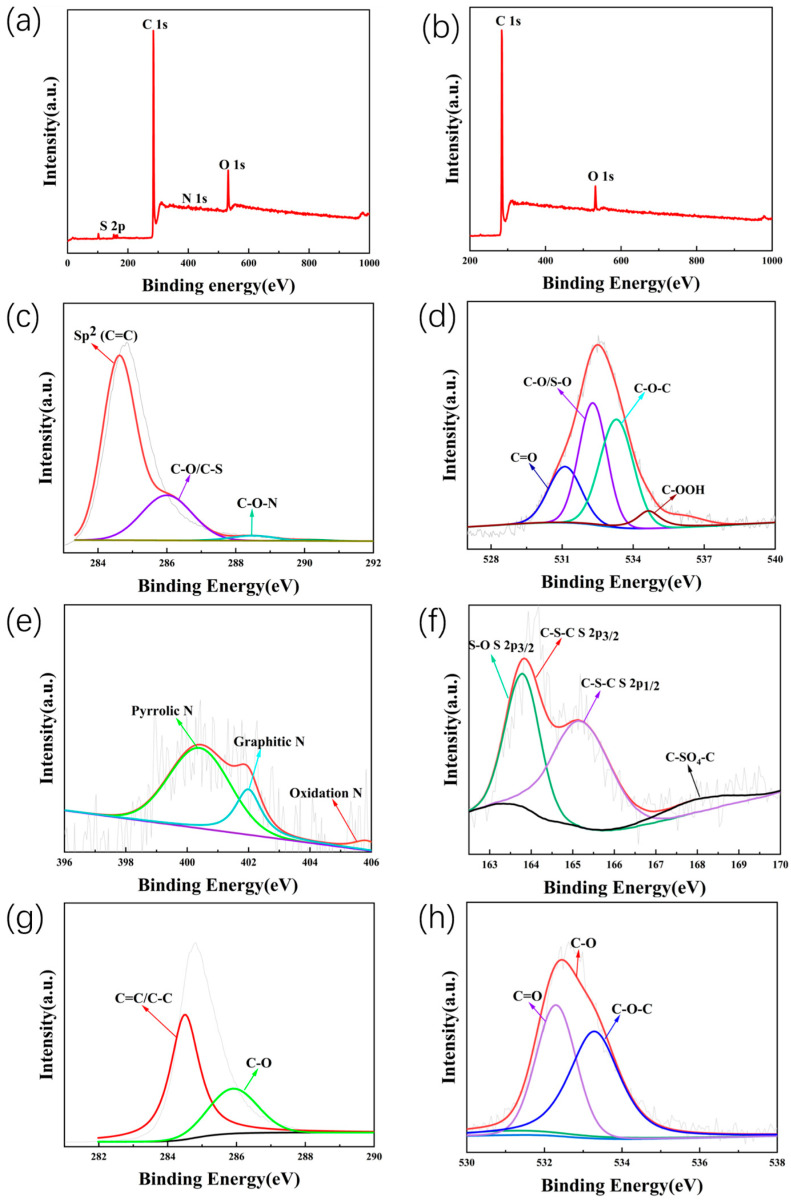
Survey XPS spectrum of (**a**) (N, S)-C sample and (**b**) OC sample; (**c**) C 1s, (**d**) O 1s, and (**e**) N 1s; (**f**) S 2p core level spectra of N, S-C, (**g**) C 1s, and (**h**) O 1s core levels of OC.

As shown in Figure 4, the XPS technique was used to investigate the surface composition of (N, S)-C. In the survey spectrum (Figure 4a), there are four stronger peaks at about 163.5, 284.5, 400.8, and 532.7 eV, which belong to C 1s, O 1s, N 1s, and S 2p, respectively, consistent with the EDS elemental mapping results. More noticeably, according to the XPS data, NSC1150 is most successful when co-doped with N, S. The atomic of N is 1.16%, and S is 1.7%. The XPS data for the other materials are available in the Supplementary Material, Table S2. However, as shown in Figure 4b), OC lacks N and S elements and only contains C and O elements. This shows that N and S are successfully doped into (N, S)-C. Figure 4c illustrates that the spectra of C 1 s in (N, S)-C1150 are located at 284.54 eV, 286.6 eV, and 288.13 eV corresponding to C=C, C-O/C-S, and C-O-N, respectively. The sp^2^ of C=C is the highest in (N, S)-C, and similarly, the most abundant in OC samples is C=C, as shown in Figure 4g [35]. Because of the formation of C-S and C-O-N, this is further evidence that N and S heteroatoms were successfully doped into the OC. After successful doping with N and S, it can create more defects, increase layer spacing, and contribute to the enhancement of the redox reaction rate during the reaction [36]. The four O 1s lines of the (N, S)-C sample (Figure 4d) are located at 531.12, 532.29, 533.29, and 536.09 eV, which are attributed to O=C, S-O/O-C, C-O-C, and C-OOH, respectively. Compared with the O 1s of OC (Figure 4h), C-OOH occurs due to the hydrothermal reaction. More importantly, a higher ratio of the atoms of C-O promotes oxidation on the surface of carbon materials, which then enhances the capacity of SIBs [37]. Figure 4e shows the N 1s XPS of the N, S-C samples, which can be divided into three lines at 400.80, 402.91, and 405.21 eV, corresponding to pyrrole-N, graphite-N, and oxidation-N, respectively. As shown by the disappearance of the pyridine nitrogen (N-6) after N and S co-doping, it can be interpreted as a possibility that pyridinium nitrogen-like nitrogen may be converted to quaternary nitrogen during the doping process [38]. Meanwhile, graphite-N increases the electrical conductivity of the material. All these factors contribute to a greater capacity and longer life cycle. Figure 4f shows the S 2p spectrum in the N, S-C sample with a binding energy of 163.8 eV for S 2p_3/2_ and 165.0 eV for S 2p_1/2_, which are attributed to C-S-C and C=S bonds. The third peak centered at 168.7 eV belongs to C-SO_4_-C. S is mainly located at the edges or defective parts of the carbon, which can effectively expand the interlayer distance and provide more pathways for Na^+^ to achieve rapid insertion and extraction, and it also effectively slows down the problem of volume expansion [27]. The synergistic effect of (N, S) co-doping alters the OC’s physical and chemical properties, which improves the electrochemical properties of carbons [39].

**Figure 5:**
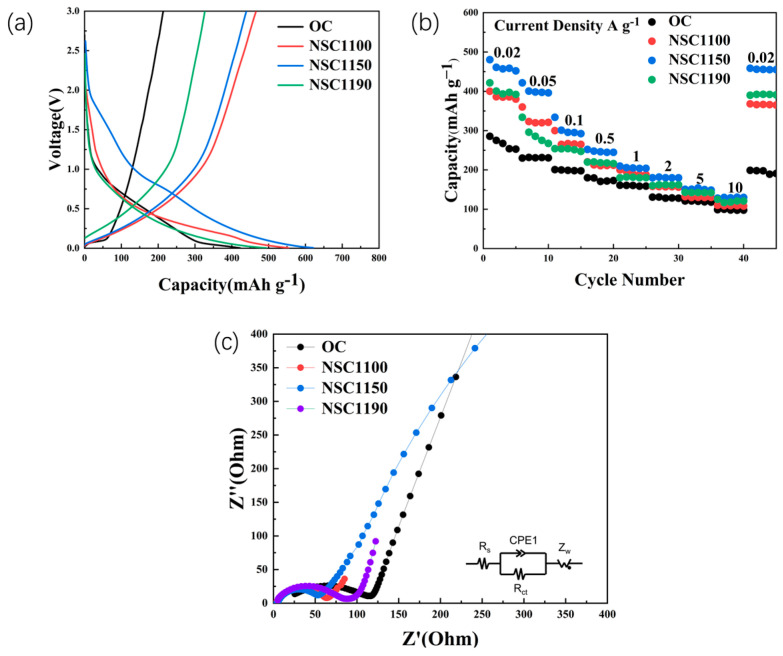
(**a**) The first discharge/charge of OC and (N, S)-C, (**b**) rate capability of sample electrodes from 20 mA g^−1^ to 10 A g^−1^, and (**c**) electrochemical impedance spectroscopy (EIS) before cycling.

The first cycle of the four samples is seen in Figure 5a. The best electrochemical performance of (N,S)-C1150 was obtained at 50 mA g^−1^, with a maximum capacity of 620 mAh g^−1^. The capacities of (N, S)-C1100 and (N, S)-C1190 were markedly higher than those of OC, the excellent capacity of which can be attributed to the contribution of the C-S and C-O-N bonding. To further explain the electrochemical properties, Figure 5b displays the rate capabilities of four samples, and the current densities ranging from 20 mA g^−1^ to 10 A g^−1^. The (N, S)-C1150 average specific capacities were 477, 423, 352, 260, 238, 201, 170, and 153 mAh g^−1^ at 0.02, 0.05, 0.1,0.5, 1, 2, 5, and 10 A g^−1^, respectively. The discharge capacities of the rate performance of OC were 296, 235, 204, 180, 168, 150, 130, and 104 mAh g^−1^ at 0.02, 0.05, 0.1,0.5, 1, 2, 5, and 10 A g^−1^, respectively. When the density was restored to 0.02 mA g^−1^, the reversible capacity of the (N, S)-C1150 was restored to more than 90% of the initial. It was shown that N and S co-doping could create a synergistic effect, which not only implies outstanding reversibility and structural stability but also greatly improved electrochemical performance. It can improve the Na^+^ mobility and electronic conductivity and promote the rapid migration and transfer of ions and electrons to create excellent multiplicity and cycling performance of (N, S)-Cs [40]. The Nyquist plots of the OC and (N, S)-C in the original state are displayed in Figure 5c. Based on the XPS data, the (N, S)-C1150 sample had more N/S content, a larger specific surface area, and a large number of edges, which indicates that the electrochemical performances are superior to that of the other materials, with a minimum EIS of only 50 Ω. The semicircle diameters in the high-frequency region were small, indicating easier charge transfer. It is worth noting that the impedances of the carbon materials doped with N and S were all less than 100 Ω, indicating that N and S doping can increase the conductivity of the materials and reduce the obstruction of Na^+^ transport. The specific resistance data are shown in Table 2 The above experimental results show that the (N, S)-C materials have good reversible capacity and Na storage performance, and (N, S)-C1150 has the best performance.

**Figure 6:**
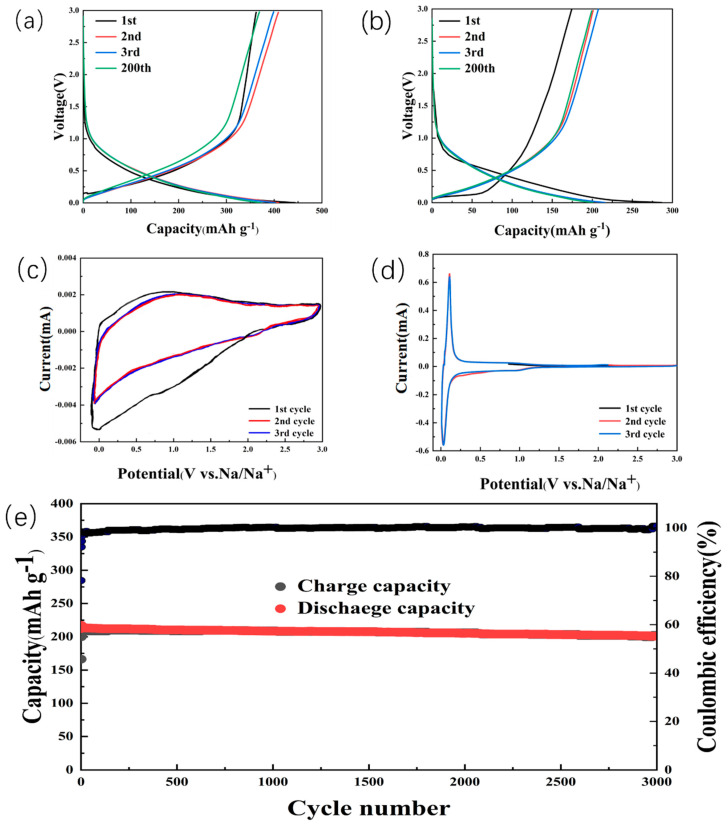
Cycling performance of (**a**) (N, S)-C and (**b**) OC at 500 mA g^–1^, CV curves of (**c**) (N, S)-C, and (**d**) OC, and (**e**) long-term cycling performance of (N, S)-C at 1A g^−1^.

Figure 6a displays the charge/discharge curves of (N, S)-C1150, and Figure 6b shows OC sodium-ion half-cells, with a current density of 500 mA g^−1^ and a potential window of 0.01–3 V. The highest capacity of (N, S)-C1150 is 440 mAh g^−1^, the capacity is 370 mAh g^−1^ after 200 cycles, and the initial Coulombic efficiency is 81%. The highest capacity of OC is 284 mAh g^−1^, and after 200 cycles, it is 218 mAh g^−1^. N and S doping carbon as an anode for SIBs has been reported by other groups (Table S1 in Supporting Information) [41,42,43,44]. One may see that the data reported in the current work are rather superior.

As shown in Figure 6c, CV curves were obtained over a potential range of 0.01 to 3 V, and the scan rate was 0.1 mV. It is known that the storage mechanism of N, S-C involves the insertion/extraction of Na^+^ [45]. The monotonically inclined curve in the high potential region corresponds to the insertion/extraction of Na ions between the carbon layers, whereas the low potential plateau corresponds to Na^+^ insertion into the pores or edges. In addition, there is a relatively strong reduction peak at 0.1 V and 0.8 V due to the decomposition of the electrolyte and the irreversible reaction between Na^+^ and C-OOH. It can form a solid electrolyte interface (SEI), which creates an irreversible peak. In the second and third cycles, the CV curves almost overlapped each other, indicating that the SEI formed only in the first cycle and stabilized in subsequent cycles. This is why it is important to show the good cycling performance of (N, S)-C1150 [46]. Figure 6d shows the CV of OC showing a pair of sharp cathodic and anodic peaks around 0.3 V due to the insertion/extraction of Na into the OC. In addition, an irreversible weak reduction peak appears at 0.3 V because of SEI film generation [43,47]. Long cycling is another important condition for the practical application of SIBs. As Figure 6e shows, even at 1 A g^−1^, the capacity is close to 220 mAh g^−1^. More importantly, the capacity degradation is less than 0.003% per cycle for 3000 cycles, and the Coulombic efficiency is close to 100%.

## 3. Experimental Section

### 3.1. Production of Origin Carbon (OC) and (N, S)-C

Corn starch was provided from the local market (Xiamen, China), and thiourea was purchased from Shanghai Aladdin Biochemical Technology Co. (Shanghai, China). First, the starch was placed in a tube furnace at 1100, 1150, and 1190 °C for carbonization under a flow of N_2_ for 2 h at a heating rate of 5 °C min^−1^ to obtain the preprocessed material. After mechanically milling at 500 r min^−1^ for 6 h, the powder obtained could pass through 300 mesh sieves. Then, 1 g of preprocessed material was dispersed into 3 mL of nitric acid, added to 100 mL of deionized water by stirring vigorously, and then transferred to a 100 mL Teflon-lined autoclave. After being heated in a blast drying oven at 180 °C for 6 h, it was washed with deionized water and alcohol several times until it was neutral and then dried in an oven at 100 °C overnight to obtain OC. The OC and thiourea were placed in a ball mill for 6 h at a mass ratio of 1:3, and the obtained mixed powders were placed in a tube furnace at 1100, 1150, and 1190 °C for carbonization under N_2_ for 2 h at a heating rate of 5 °C min^−1^. The obtained (N, S)-C was then noted as (N, S)-C1100, (N, S)-C1150, and (N, S)-C1190.

### 3.2. Sample Characterization

The microstructures and morphologies of the as-prepared samples were characterized via scanning electron microscopy (SEM, LEO1530 VP, Freising, Germany) and high-resolution transmission electron microscopy (HRTEM, TECNAI F30). The crystallographic information was investigated using powder X-ray diffraction (XRD, Rigaku Ultima IV X-ray diffractometer with Cu Kαradiation (λ = 1.54056 Å)). The specific surface area and pore size distribution were measured via N_2_ adsorption/desorption experiments at 77 K using a Micromeritics TriStar II3020 surface area and pore analyzer. The Raman spectra of the as-prepared samples were recorded on an XploRA confocal Raman microscope (Jobin Yvon-Horiba, Longjumeau, France) with an excitation wavelength of 532 nm.

### 3.3. Electrochemical Measurements

The working electrodes were prepared by mixing the active material, Super P, and PVDF binders at a weight ratio of 8:1:1 in N-methyl-2-pyrrolidinone (NMP) solvent to obtain a homogeneously dispersed slurry. The slurry was then coated on Cu or Al foil for the anode and cathode, respectively, and then dried at 120 °C under vacuum overnight. The mass of the active material was about 1 mg cm^−2^, and 60 μL of 1 M NaPF6 in diglyme was used as the electrolyte. Half cells were assembled in an argon-filled glovebox (H_2_O < 1 ppm, O_2_ < 1 ppm) using CR2016-type coin cells with a sodium foil as the counter electrode, a glass fiber as the separator, 1 M NaPF_6_ in diglyme as an electrolyte and the slurry-coated Cu foil as a working electrode.

The discharge/charge tests were carried out on a Neware battery measurement system (CT4000, China) over a voltage range of 0.01–3 V (vs. Na^+^/Na) at various current densities. An electrochemical workstation (CHI660D, Shanghai Chenhua, Shanghai, China) was used to acquire the cyclic voltammetry (CV) with a scan rate of 0.1 mV s^−1^ from 0.01 to 3.00 V. The electrochemical impedance spectroscopy (EIS) was measured using DH7000C (DongHua Analytical) with a frequency range from 0.01 Hz to 100 KHz.

## 4. Conclusions

The (N, S)-C co-doped carbons were successfully produced from low-cost corn starch. It can be seen that the capacity of N and S co-doped carbon material with a high specific surface area significantly increased. It not only broadens the layer spacing, reducing the resistance of Na^+^ diffusion, but also provides active sites for Na^+^ due to the different electronegativity of N and C. The maximum capacity was reported for the (N, S)-C1150 electrode, which exhibited 620 mAh g^−1^ at 50 mA g^−1^ and approached 400 mA g^−1^ after 200 cycles at a current density of 500 mA g^−1^. Even at a high rate of high current of 10 A g^−1^, the capacity reached 170 mAh g^−1^. More importantly, after 3000 charge/discharge cycles at 1 A g^−1^, the capacity decay was less than 0.003% per cycle. Due to the simplicity and low cost, the reported method can be used for large-scale production of co-doped carbonous materials for SIBs.

## Figures and Tables

**Table 1:** Physical parameters for (N, S)-C and OC.

Sample	I_D_/I_G_	S_BET_(m^2^ g^−1^)	d_002_(nm)	D_P_(nm)
NSC1100	1.03	563.45	0.381	6.25
NSC1150	1.21	683.85	0.386	3.99
NSC1190	0.94	296.17	0.376	3.64
OC	0.86	315.86	0.360	7.75

S_BET_—BET surface area. D_P_—pore diameter.

**Table 2:** EIS data interpretation of the four samples.

Material	Rs	Rct
NSC-1100	3.48	70.53
NSC-1150	3.32	47.6
NSC-1190	6.35	95.7
OC	8.92	120.4

## Data Availability

Not applicable.

## Supplementary Materials

The following supporting information can be downloaded at: https://www.mdpi.com/article/10.3390/molecules28217314/s1. Figure S1: (a) SEM images of OC (b) the EDS of OC, (c, d) TEM images of OC respectively; Table S1: Comparison of the electrochemical performance of N, S co-doped carbon with other car-bon materials reported in previous literature; Table S2: Comparison of the electrochemical performance of N, S co-doped carbon with this work.
