# Supplementary material for: Synthesis of Low-Cost and High-Performance Dual-Atom Doped Carbon-Based Materials with a Simple Green Route as Anodes for Sodium-Ion Batteries
Source: Molecules. 2023 Oct 28;28(21):7314. doi: 10.3390/molecules28217314 (PMC10649136; doi:10.3390/molecules28217314)
Supplement: Supplementary file 1 [file molecules-28-07314-s001.zip › molecules-2672282-supplementary.pdf]

Supplementary Materials

Synthesis of Low-Cost and High-Performance Dual-Atom Doped Carbon-Based Materials with a Simple Green Route as Anodes for Sodium-Ion Batteries

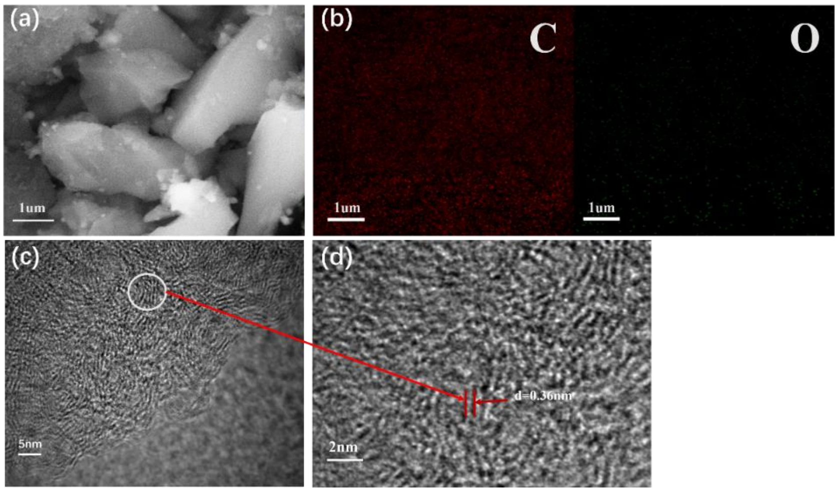

Figure S1 (a) SEM images of OC (b) the EDS of OC, (c, d) TEM images of OC respectively.

Table S1 Comparison of the electrochemical performance of N, S co-doped carbon with other carbon materials reported in previous literature.

Precursor	Capacity and cycle stability	High-Rate Capability (mAh g^{-1})	ICE	Ref
This work	183 mAh g^{-1} after 3000 cycles at 1A g^{-1}	170 mAh g^{-1} at 10 A g^{-1}	81%	
Bagasse	155 mAh g^{-1} after 2000 cycles at 1 A g^{-1}	148 mAh g^{-1} at 5 A g^{-1}	58.7%	33
$SnCl_4{\cdot}5H_2O$	380.1 mAh g^{-1} after 200 cycles at 500 mA g^{-1}	310.6 mAh g^{-1} at 4 A g^{-1}	68.9%	36
algae-Carrageen	227 mAh g^{-1} after 100 cycles at 0.1 A g^{-1}	109 mAh g^{-1} at 10 A g^{-1}	32.7%	39
donkey-hide gelatin pulp	266 mAh g^{-1} after 500 cycles at 0.5 A g^{-1}	98 mAh g^{-1} at 5 A g^{-1}	62%	41
citrate sodium	223 mAh g^{-1} after 2000 cycles at 1 A g^{-1}	102 mAh g^{-1} at 10 A g^{-1}	/	42
Mango-peels	351 mAh g^{-1} after 200	136 mAh g^{-1} at 4 A	52.03%	43

medicine residue	cycles at 100 mA g^{-1}<br>801 mAh g^{-1} at 0.1A g^{-1}	g^{-1}<br>402 mAh g^{-1} at 5 A g^{-1}	/	44

Table S2 Comparison of the electrochemical performance of N, S co-doped carbon with this work

**Sample**	**C**	**O**	**N**	**S**
**NSC1100**	92.09%	5.39%	1.08%	1.44%
**NSC1150**	87.92%	9.22%	1.16%	1.7%
**NSC1190**	92.04%	6.07%	0.78%	1.11%
